# Examining the patient profile and variance of management and in‐hospital outcomes for Australian adult burns patients

**DOI:** 10.1111/ans.17985

**Published:** 2022-08-22

**Authors:** Lincoln M. Tracy, Anne Darton, Belinda J. Gabbe, Kathryn Heath, Rochelle Kurmis, Carl Lisec, Cheng Lo, Yvonne Singer, Fiona M. Wood, Heather J. Cleland

**Affiliations:** ^1^ School of Public Health and Preventive Medicine Monash University Melbourne Victoria Australia; ^2^ Statewide Burn Injury Service New South Wales Agency for Clinical Innovation Sydney New South Wales Australia; ^3^ Health Data Research UK, Swansea University Medical School Swansea University Swansea UK; ^4^ Adult Burns Service Royal Adelaide Hospital Adelaide South Australia Australia; ^5^ Faculty of Medicine The University of Queensland Brisbane Queensland Australia; ^6^ Professor Stuart Pegg Adult Burns Centre The Royal Brisbane And Women's Hospital Brisbane Queensland Australia; ^7^ Victorian Adult Burns Service The Alfred Melbourne Victoria Australia; ^8^ Department of Surgery, Central Clinical School Monash University Melbourne Victoria Australia; ^9^ State Adult Burn Unit Fiona Stanley Hospital Murdoch Western Australia Australia; ^10^ Burn Injury Research Unit University of Western Australia Perth Western Australia Australia

**Keywords:** adult, Australia, burn, registry, variation

## Abstract

**Background:**

Burn injuries are a common subtype of trauma. Variation in models of care impacts clinical measures of interest, but a nation‐wide examination of these measures has not been undertaken. Using data from the Burns Registry of Australia and New Zealand (BRANZ), we explored variation between Australian adult burn services with respect to treatment and clinical measures of interest.

**Methods:**

Data for admissions July 2016 to June 2020 were extracted. Clinical measures of interest included intensive care admission, skin grafting, in‐hospital death, unplanned readmissions, and length of stay (LOS). Estimated probabilities, means, and corresponding 95% confidence intervals (CI) were calculated for each service.

**Results:**

The BRANZ recorded 8365 admissions during the study period. Variation between specialist burn services in admissions, demographics, management, and clinical measures of interest were observed. This variation remained after accounting for covariates. Specifically, the adjusted proportion (95% CI) of in‐hospital mortality ranged from 0.15% (0.10–0.21%) to 1.22% (0.9–1.5%). The adjusted mean LOS ranged from 3.8 (3.3–4.3) to 8.2 (6.7–9.7) days.

**Conclusions:**

A decade after its launch, BRANZ data displays variation between Australian specialist burn services. We suspect differences in models of care between services contributes to this variation. Ongoing research has begun to explore reasons underlying how this variation influences clinical measures of interest. Further engagement with services about models of care will enhance understanding of this variation and develop evidence‐based guidelines for burn care in Australia.

## Graphical abstract

A decade after its launch, data from the Burns Registry of Australia and New Zealand displays variation between specialist burn services in Australia due to differences in models of care. Ongoing research has begun to explore reasons underlying variation in models of care influences outcomes.

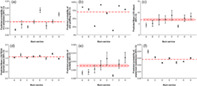

## Introduction

Burn injuries are a global health problem. The World Health Organization reported nearly 11 million people worldwide were burned severely enough to require medical attention in 2004.^1^ More than 2500 people are admitted to an Australian specialist burn service each year.^2^ Despite their relative scarcity in comparison to hospitalisations for other injuries, burns are a complex subset of trauma associated with high personal and financial costs.^3^ Many patients require a protracted period of surgical, medical, physical, and psychological rehabilitative measures that can span decades.^3^, ^4^, ^5^

Benchmarking clinical performance is becoming an increasingly popular quality improvement tool in healthcare. Clinical quality registries are a key contributor to this surge in popularity.^6^ A key component of benchmarking is comparing care providers against their peers or a recommended standard. Multiple national and international burn registries exist.^7^, ^8^, ^9^ Despite the vast amount of research relating to data held within these registries, there is limited research focusing on variation in practice and benchmarking burn care.^10^, ^11^, ^12^, ^13^

In 2016, Cleland and colleagues determined the variation between participating services in the treatment of patients and specific in‐hospital clinical measures of interest following the launch of the Burns Registry of Australia and New Zealand (BRANZ).^14^ This provided an initial overview of variation in practice in Australian and New Zealand burn care. However, the registry has developed and expanded since then.^15^ Most importantly, all 17 specialist services now contribute data to the BRANZ. There has yet to be an investigation of the patients and management approaches of services since these data became available. The aim of our study was to use the first four years of BRANZ data with all Australian specialist adult burn services contributing, and to highlight specific areas of practice where there is variation in practice between services that may affect treatment efficacy.

## Methods

### Setting and participants

This study focused on the eight Australian specialist adult burn services. Data for adult patients (≥16 years) registered by the BRANZ and admitted July 2016–June 2020 were extracted. Patients treated at a New Zealand burn service, patients with an unknown date of injury, adult patients treated at a paediatric hospital, patients with an inhalation injury but no cutaneous burn, patients deemed to have non‐survivable injures on arrival and treated with palliative intent, and intersex patients or patients of indeterminate gender were excluded.

### Data management and analysis

Demographic, burn cause, injury severity, surgical management, and in‐hospital data were extracted. Data management details can be found in Document S1. Data are presented by contributing burn service. Summary statistics (frequencies and percentages for categorical variables, medians and interquartile ranges [IQR] for continuous variables due to the skewed nature of the data) described cases. Differences between patients at each service were assessed using chi‐square (categorical variables) or Kruskal Wallis tests (continuous variables). *Post hoc* Dunn's test of multiple comparisons and pairwise chi‐square tests assessed differences between individual services. Bonferroni corrections were applied to *post hoc* tests to minimize the false positive rate.

Variation in practice between burn services was assessed with multivariable, mixed‐effects linear and logistic regression modelling. The contributing burn service was treated as a random effect to account for the correlation between cases within each service. The fixed effects were the covariates describing the difference in the case mix between the contributing services known to affect the clinical measures of interest. The selected covariates were: percentage total body surface area (TBSA) burned, maximum recorded burn depth, age, gender, the presence of an inhalation injury, the primary cause of the injury, and whether special body area (i.e., face, hands, feet, or genitals/perineum) was affected. Mixed‐effects logistic regression modelling was used for intensive care unit (ICU) admission, skin grafting, in‐hospital mortality, and unplanned readmissions due to complications. From these models, adjusted proportions and 95% confidence intervals (CIs) for each clinical measure of interest was calculated for each service. The output of the regression models are presented in Tables [Link], [Link]. Due to the skewed nature of the data, hospital length of stay (LOS) and LOS/TBSA burned data were logarithmically‐transformed and analysed with mixed effects linear regression. The adjusted mean hospital LOS and LOS/TBSA for each service was calculated after back‐transformation. Pairwise differences in adjusted proportions and means were assessed using one‐way analyses of variance with Bonferroni corrections. Pairwise comparison outputs are presented in Tables [Link], [Link]. Data manipulation and statistical analyses were performed using Stata Version 14 (StataCorp, USA); *p*‐values < 0.05 were statistically significant. Figure production details can be found in Document S1. Ethics approval for the registry and study was obtained from the Monash University Human Research Ethics Committee (reference CF08/2431‐2008001248). Readmission data were not available for service A; this service is excluded from these analyses.

## Results

### Patient profile

There were 8365 admissions meeting inclusion criteria (Fig. S1). A description of the patient population and variation between services can be found in Document S2. The rate of missing data for key confounders and clinical measures of interest (including burn injury cause, %TBSA, inhalation injury, skin grafting, and ICU admission) was very low (Table 1) and there was no clear pattern of missingness between centres.

**Table 1:** Profile of cases managed at each Australian adult burn service

	A (*n* = 1599)	B (*n* = 242)	C (*n* = 1389)	D (*n* = 1039)	E (*n* = 945)	F (*n* = 307)	G (*n* = 1496)	H (*n* = 1348)	*p*
Age, median (IQR) years	38 (27‐54)	40 (26‐55)	42 (29‐57)	40 (26‐56)	41 (27‐57)	36 (25‐50)	40 (26‐54)	42 (28‐58)	<0.001
Male	1106 (69.2%)	146 (60.3%)	918 (66.1%)	757 (72.9%)	659 (69.7%)	224 (73.0%)	1092 (73.0%)	980 (72.7%)	<0.001
Primary cause^a^									<0.001
Flame	576 (36.2%)	103 (42.7%)	472 (34.0%)	411 (39.7%)	407 (43.2%)	117 (38.2%)	603 (40.6%)	783 (58.3%)	
Scald	483 (30.4%)	47 (19.5%)	543 (39.1%)	244 (23.6%)	278 (29.5%)	62 (20.3%)	342 (23.0%)	340 (25.3%)	
Contact	292 (18.4%)	58 (24.1%)	215 (15.5%)	186 (18.0%)	112 (11.9%)	41 (13.4%)	349 (23.5%)	114 (8.5%)	
Other cause	240 (15.1%)	33 (13.7%)	158 (11.4%)	195 (18.8%)	146 (15.5%)	86 (28.1%)	190 (12.8%)	105 (7.8%)	
TBSA, median (IQR) %^b^	1.6 (0.5‐4.3)	1.8 (0.5‐5.5)	2.5 (1.0‐5.0)	2.0 (1.0‐6.0)	4.0 (1.0‐10.0)	3.0 (1.0‐6.0)	2.5 (1.0‐8.0)	4.0 (2.0‐9.0)	<0.001
Major burns^b^	56 (3.6%)	12 (5.1%)	58 (4.2%)	60 (5.8%)	85 (9.0%)	11 (4.2%)	117 (7.8%)	118 (8.8%)	<0.001
Inhalation injury^c^	12 (0.8%)	11 (4.5%)	21 (1.5%)	40 (3.9%)	63 (6.7%)	16 (5.3%)	115 (7.7%)	58 (4.3%)	<0.001
Deepest skin layer affected^d^									<0.001
Superficial dermal	22 (1.4%)	23 (10.5%)	336 (24.6%)	46 (4.4%)	52 (5.5%)	29 (11.6%)	296 (19.9%)	316 (23.5%)	
Mid dermal	456 (29.7%)	22 (10.0%)	509 (37.2%)	184 (17.7%)	263 (28.0%)	61 (24.5%)	8 (0.5%)	300 (22.3%)	
Deep dermal	738 (48.1%)	62 (28.2%)	150 (11.0%)	530 (51.0%)	246 (26.2%)	73 (29.3%)	173 (11.7%)	369 (27.4%)	
Full thickness	317 (20.7%)	113 (51.4%)	372 (27.2%)	279 (26.9%)	378 (40.3%)	86 (34.5%)	1007 (67.9%)	360 (26.8%)	
Time from injury to admission, median (IQR) hours^e^	75.1 (23.6‐149.4)	109.8 (7.8‐322.1)	11.2 (3.0‐47.8)	91.5 (11.0‐210‐210.0)	36.0 (8.5‐202.5)	16.2 (2.7‐76.8)	79.9 (10.4‐199.8)	7.2 (2.0‐55.6)	<0.001
Referral source									<0.001
Scene of injury	347 (21.7%)	51 (21.1%)	257 (18.5%)	178 (17.1%)	88 (9.3%)	81 (26.4%)	308 (20.6%)	525 (38.9%)	
Other hospital	272 (17.0%)	35 (14.5%)	689 (49.6%)	577 (55.5%)	695 (73.5%)	53 (17.3%)	998 (66.7%)	605 (44.9%)	
Outpatients	702 (43.9%)	98 (40.5%)	33 (2.4%)	145 (14.0%)	71 (7.5%)	22 (7.2%)	39 (2.6%)	35 (2.6%)	
Other source	278 (17.4%)	58 (24.0%)	410 (29.5%)	139 (13.4%)	91 (9.6%)	151 (49.2%)	151 (10.1%)	183 (13.6%)	
Surgical management^f^	1281 (80.1%)	188 (77.7%)	1156 (83.2%)	899 (86.5%)	695 (73.8%)	284 (93.1%)	1188 (79.4%)	960 (71.2%)	<0.001
Skin graft^†^ ^,g^	1082 (84.6%)	145 (77.1%)	303 (26.2%)	825 (91.7%)	457 (65.7%)	39 (13.7%)	1051 (88.5%)	736 (77.5%)	<0.001
ICU admission^h^	62 (3.9%)	18 (7.4%)	81 (5.9%)	101 (9.7%)	178 (18.8%)	20 (6.6%)	148 (9.9%)	209 (15.5%)	<0.001
Mechanically ventilated in ICU^‡^ ^,i^	35 (71.4%)	16 (88.9%)	57 (70.4%)	73 (72.3%)	148 (83.1%)	9 (45.0%)	130 (87.8%)	176 (84.2%)	<0.001
Time ventilated in ICU, median (IQR) hours^j^	59.0 (20.0‐168.0)	59.0 (27.0‐194.0)	35.9 (15.3‐156.0)	59.7 (21.3‐170.0)	32.1 (17.9‐122.8)	19.5 (13.0‐66.0)	157.5 (41.0‐290.5)	62.0 (19.0‐201.8)	<0.001
LOS, median (IQR) days	3.7 (1.5‐7.3)	3.0 (1.1‐10.9)	3.8 (2.0‐7.6)	2.3 (0.4‐9.2)	3.9 (1.1‐13.2)	3.8 (1.5‐7.9)	6.1 (2.0‐11.9)	7.6 (3.1‐13.8)	<0.001
LOS/TBSA, median (IQR) days^k^	1.7 (0.8‐3.8)	1.8 (0.7‐3.7)	1.4 (0.8‐3.2)	0.8 (0.4‐2.0)	0.9 (0.3‐2.8)	1.0 (0.5‐2.6)	1.7 (0.8‐3.9)	1.6 (0.9‐3.3)	<0.001
Discharge disposition^l^									<0.001
Home/usual residence	1486 (92.9%)	209 (86.4%)	1281 (92.2%)	972 (93.6%)	748 (79.4%)	240 (78.2%)	1294 (86.6%)	1112 (82.6%)	
Other hospital	69 (4.3%)	14 (5.8%)	71 (5.1%)	43 (4.1%)	95 (10.1%)	25 (8.1%)	82 (5.5%)	189 (14.0%)	
Died	6 (0.4%)	< 5	< 5	9 (0.9%)	< 5	1 (0.3%)	10 (0.7%)	19 (1.4%)	
Other disposition	38 (2.4%)	18 (7.4%)	33 (2.4%)	14 (1.3%)	88 (9.3%)	41 (13.4%)	109 (7.3%)	27 (2.0%)	
Unplanned readmission^m^	NA	12 (5.0%)	46 (3.3%)	21 (2.0%)	28 (3.0%)	8 (2.6%)	32 (2.1%)	42 (3.1%)	0.09

*Note*: Data presented as frequency (percentage) unless otherwise specified.

Abbreviations: ICU, intensive care unit; IQR, interquartile range; LOS, length of stay; NA, not available; TBSA, total body surface area.

^†^ Percentage relative to number of patients who underwent a burn wound management procedure in theatre.

^‡^ Percentage relative to the number of patients admitted to the ICU.

Data missing/unknown for ^a^34 patients, ^b^111 patients, ^c^54 patients, ^d^189 patients, ^e^27 patients, ^f^5 patients, ^g^12 patients, ^h^29 patients, ^i^13 patients, ^j^36 patients, ^k^129 patients, ^l^6 patients, and ^m^1 patient.

### ICU admission

**Fig. 1:**
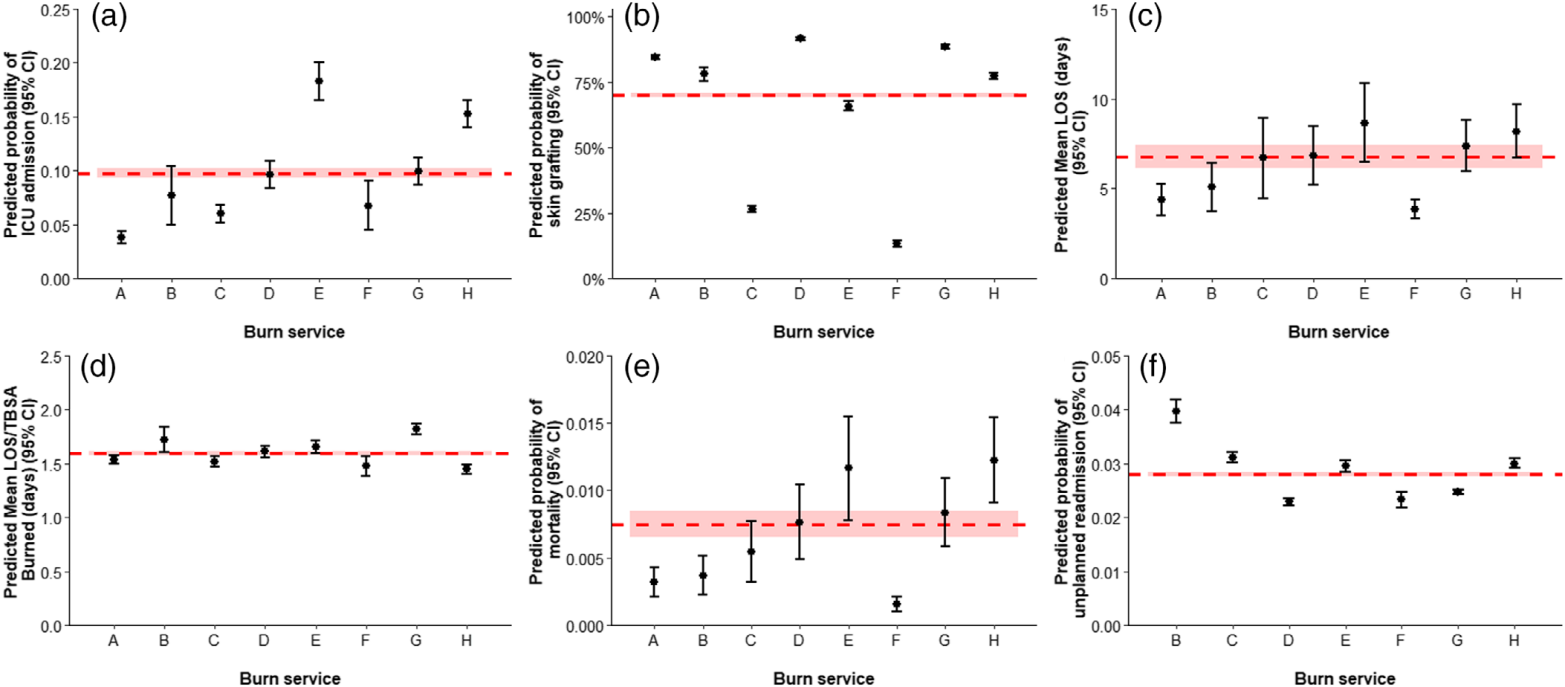
(a) Adjusted proportion of patients admitted to the intensive care unit (ICU); (b) adjusted proportion of patients undergoing skin grafting; (c) adjusted mean length of stay (LOS), (d) adjusted mean LOS per percentage of total body surface area (TBSA) burned, (e) adjusted proportion of in‐hospital mortality, and (f) adjusted proportion of patients experiencing unplanned readmission. The letters A‐H represent the eight Australian specialist burn services that treat adult patients. Readmission data were not available for service a. Error bars represent 95% confidence intervals (CI). The red dotted line represents the overall proportion/mean for the whole sample; the red shading represents the overall 95% CI. The adjusted proportions and means account for the random effect of contributing burn service and the fixed effects of the following covariates: Percentage total body surface area, maximum recorded burn depth, age, gender, the presence of an inhalation injury, the primary cause of the injury, and whether special body area (i.e., face, hands, feet, or genitals/perineum) was affected.

The ICU admission rate differed between services (3.9%–18.8%; Table 1). Services E and H had greater proportion of patients admitted to ICU compared to others, with E being greater than H. The proportion of patients admitted to ICU increased over time for all services except service C (Fig. S2a). Services E and H had a greater adjusted proportion patients admitted to ICU (18.3%, 95% CI 16.6%–20.0% and 15.3%, 95% CI 14.0%–16.5%) compared to other services (range 3.7%–9.9%; Fig. 1a), but E and H did not differ.

### Skin grafting

Most patients underwent a burn wound management procedure in theatre (Table 1). Service F had a greater proportion of patients undergoing a burn wound management procedure. Of the patients taken to theatre, over two‐thirds (69.9%) received a skin graft. The proportion of patients receiving a skin graft varied between services (13.7%–91.7%). Services C and F had a smaller proportion of patients who received a skin graft compared to the others, with F being smaller than C. Skin grafting rates were consistent over time (Fig. S2b). After accounting for confounding factors, services C and F had a lower adjusted proportion of patients receiving a skin graft compared to others, with F being smaller than C (Fig. 1b).

### LOS

The median (IQR) LOS ranged from 2.3 (1.1–13.2) days to 7.6 (3.1–13.8) days (Table 1). Services G and H had longer median hospital LOS compared to others. Within individual services, the median LOS remained consistent over time (Fig. S2c). Service A had a shorter adjusted mean LOS compared to services E and H (Fig. 1c). The adjusted mean LOS did not vary substantially after including ICU admission as an additional covariate (Fig. S3a).

### LOS/TBSA burned

The median (IQR) LOS/TBSA burned ranged from 0.8 (0.4–2.0) days to 1.8 (0.7–3.7) days (Table 1). Services A, B, C, G, and H had greater LOS/TBSA compared to services D, E, and F. Within individual services, the median LOS/TBSA burned remained consistent over time (Fig. S2d). Service G had a greater adjusted mean LOS/TBSA than all other services except for service B. Service B had a greater adjusted mean LOS/TBSA compared to services C and H (Fig. 1d). Service H had a smaller adjusted mean LOS/TBSA compared to services D and E.

### In‐hospital mortality

Less than 1 % of patients died during their admission. All services recorded at least one death (service‐level mortality rate range 0.3–1.2%; Table 1). Annual in‐hospital mortality rates varied over time (Fig. S2e). Service H had a greater adjusted proportion of in‐hospital mortality compared to services A, C, and F (Fig. 1e). Service E also had a greater adjusted proportion of in‐hospital mortality compared to services A and C. The adjusted proportion of in‐hospital mortality did not vary substantially after including ICU admission as an additional covariate (Fig. S3b).

### Unplanned readmissions

Unplanned readmissions were rare, occurring in 2.3% of surviving patients. The proportion of patients experiencing an unplanned readmission ranged from 2.0% (service D) to 5.0% (service B); this difference was not significant (Table 1). Year‐by‐year readmission rates increased for services G and H (Fig. S2f). Service B had the highest adjusted proportion of patients experiencing an unplanned readmission (Fig. 1f). The adjusted proportion of unplanned readmissions did not vary substantially after including ICU admission as an additional covariate (Fig. S3c).

## Discussion

This study highlights the disparity between demographics, injury characteristics, and clinical measures of interest of adult patients treated at Australian burn services. However, the observed differences in casemix cannot fully explain the variation in the measures of interest, as it remains after controlling for clinically relevant demographic and injury factors via multivariable, mixed‐effects regression modelling. Therefore, this variation may exist because of differences in clinical models of care between specialist burn services.

The number of patients admitted to specialist Australian burn services differed over the four‐year study period. Geographic diversity and population density may contribute to this variation. Jurisdictional differences in the proportion of burn‐related deaths in the pre‐hospital and hospital environment exist; geographically smaller jurisdictions have a greater proportion of in‐hospital deaths compared to geographically larger jurisdictions.^16^ Differences in transfer availability and policies may also contribute. Services that receive and manage all burns patients within their jurisdiction—regardless of whether they meet national referral criteria—will have more admissions compared to services in other jurisdictions where hospitals without a specialist burn service treat patients not meeting referral criteria.

Differences in demographic and injury characteristics were observed between services. Most notably, patients at services E and H had a larger median TBSA burned compared to patients at other services. We also observed variation in the unadjusted rates of ICU admission, skin grafting, and in‐hospital mortality. Importantly, variation in these clinical measures of interest remained after accounting for differences in key factors (age, TBSA burned, burn depth, *etc*.). This suggests that how services manage patients (i.e., models of care) is a key contributor to this variation. The two services with the lowest adjusted proportion of in‐hospital mortality (A and F) have lower ICU admission rates, while the two services with the highest in‐hospital mortality proportion (E and H) had the highest ICU admission rates. This is unsurprising, as ICU admission is associated with an increased risk of mortality regardless of other factors. Adding ICU admission to the multivariable models did not substantially influence the model. Additionally, services A and F had a lower adjusted hospital LOS but significantly different adjusted proportions of skin grafting.

Clinical models of care evolve over time and are based on the assessment of the patient, the environment, and the experience of the treating team. Few services have detailed management algorithms, which results in a lack of visibility and potential inconsistency in treatment approaches within and across burn care services. A complete model of burn care, ranging from triaging and pre‐hospital management through to long‐term rehabilitation and scar management, is an incredibly broad spectrum, which requires a series of smaller focussed studies to understand variation identified in this study. We hope this paper will act as the required catalyst for further engagement with burn services to optimize impact on clinical measures of interest. Similar work in paediatric services is already underway.^17^

Since the previous paper from Cleland *et al*.,^14^ the BRANZ has undergone refinement to ensure the collected data has clinical relevance and meaning.^15^ Significant efforts have been made to provide high‐quality training to data collection and entry staff to ensure accurate data entry. This is essential if clinicians are to engage in further examining and responding to the results. One additional area where the BRANZ could improve is the collection of long‐term outcome data. The registry has obtained funding to pilot the centralized collection of patient‐reported outcomes in patients admitted to a Victorian burn service. Patient recruitment for the pilot is complete and data collection will conclude in early 2023.

This study is not without limitations. This study has only focused on Australian specialist adult burn services. These findings may therefore not translate to other settings, most notably New Zealand. In addition, this study only considered patients registered in the BRANZ. The registry should consider linkage with other data sets to capture a wider cohort of patients, which would allow for exploration of the association between in‐hospital treatment and post‐discharge outcomes, among other things. Furthermore, the BRANZ does not collect information on clinical decision‐making or infrastructure processes (staffing levels, operating theatre availability, *etc*.) that influence management and clinical measures of interest. The BRANZ can determine how long it took for debridement and grafting to occur but it does not collect information on *why* it took longer for one patient compared to another (e.g., the presence of other injuries requiring attention, surgeon beliefs about early versus delayed excision, *etc*.). In addition, the BRANZ does not collect physiological data and therefore cannot utilize disease severity systems such as the Acute Physiology and Chronic Health Evaluation II score. The inability to collect data on all potential confounders means there are factors we are unable to control for in our analyses. Furthermore, as we are using observational data, we can only describe associations within our data, rather than proving causation.

A clinical quality registry for specialist burn care in Australia has existed for over a decade. Our results demonstrate variation in practice (e.g., skin grafting) and clinical measures of interest between services arising from differences in clinical management. A more in‐depth exploration of the reasons for variation in models of care between services (e.g., Delphi methodologies, focus groups, etc.) is required and will be a key driver in improving the care provided to burns patients. Registry data can continually be used to identify areas of variation in practice, support low‐performing services in investigating local data and determining whether improvements could be made, and tracking the effects of any changes to models of care over time.

## Author contributions

**Lincoln M. Tracy:** Conceptualization; data curation; formal analysis; methodology; project administration; software; visualization; writing – original draft; writing – review and editing. **Anne Darton:** Methodology; supervision; writing – review and editing. **Belinda Gabbe:** Methodology; resources; supervision; writing – review and editing. **Kathryn Heath:** Methodology; supervision; writing – review and editing. **Rochelle Kurmis:** Methodology; supervision; writing – review and editing. **Carl Lisec:** Conceptualization; methodology; supervision; writing – review and editing. **Cheng Lo:** Methodology; supervision; writing – review and editing. **Yvonne Singer:** Methodology; supervision; writing – review and editing. **Fiona Wood:** Conceptualization; methodology; supervision; writing – review and editing. **Heather Cleland:** Conceptualization; methodology; supervision; writing – review and editing.

## Conflict of interest

None declared.

## Supporting information


**Document S1:** Supporting methods.

**Document S2:** Description of patient population and demographic, injury, and clinical measure variation between services.

**Figure S1:** Patient flow chart and characteristics.

**Figure S2:** Year‐by‐year changes for (a) ICU admissions, (b) skin grafts, (c) median LOS, (d) median LOS/TBSA burned, (e) in‐hospital mortality, and (f) unplanned readmissions at individual service level.

**Figure S3:** Regression modelling output for selected clinical measures of interest. (a) Adjusted mean length of stay, (b) adjusted proportion of in‐hospital mortality, and (c) adjusted proportion of patients experiencing unplanned readmission.

**Table S1:** Modelling output for adjusted proportion of admission to ICU.

**Table S2:** Modelling output for adjusted proportion of receiving skin graft.

**Table S3:** Modelling output for adjusted mean LOS.

**Table S4:** Modelling output for adjusted mean LOS/TBSA.

**Table S5:** Modelling output for adjusted proportion of in‐hospital mortality.

**Table S6:** Modelling output for adjusted proportion of unplanned readmissions.

**Table S7:** Pairwise comparisons for age by service.

**Table S8:** Pairwise comparisons for male gender by service.

**Table S9:** Pairwise comparisons for primary cause by service.

**Table S10:** Pairwise comparisons for TBSA burned by service.

**Table S11:** Pairwise comparisons for major burns by service.

**Table S12:** Pairwise comparisons for inhalation injury by service.

**Table S13:** Pairwise comparisons for deepest skin layer affected by service.

**Table S14:** Pairwise comparisons for time from injury to admission by service.

**Table S15:** Pairwise comparisons for referral source by service.

**Table S16:** Pairwise comparisons for mechanical ventilation in ICU by service.

**Table S17:** Pairwise comparisons for mechanical ventilation time by service.

**Table S18:** Pairwise comparisons for discharge disposition by service.

**Table S19:** Pairwise comparisons for ICU admission by service.

**Table S20:** Pairwise comparisons for adjusted proportions of patients admitted to the ICU.

**Table S21:** Pairwise comparisons for undergoing a burn wound management procedure in theatre by service.

**Table S22:** Pairwise comparisons for undergoing skin grafting by service.

**Table S23:** Pairwise comparisons for adjusted proportion of patients receiving a skin graft.

**Table S24:** Pairwise comparisons for hospital LOS by service.

**Table S25:** Pairwise comparisons for adjusted mean LOS.

**Table S26:** Pairwise comparisons for adjusted mean LOS/TBSA burned.

**Table S27:** Pairwise comparisons for LOS/TBSA by service.

**Table S28:** Pairwise comparisons for adjusted proportion of in‐hospital mortality.

**Table S29:** Pairwise comparisons for predicted probability of unplanned readmission.
