# Supplementary material for: Examining the patient profile and variance of management and in‐hospital outcomes for Australian adult burns patients
Source: ANZ J Surg. 2022 Aug 22;92(10):2641–7. doi: 10.1111/ans.17985 (PMC9804322; doi:10.1111/ans.17985)
Supplement: Supplementary file 1 — Document S1: Supporting methods. [file ANS-92-2641-s010.docx]

**Document S1 – Supporting Methods**

Eight Australian hospitals with specialist burn services managing adult patients contributed data to the Burns Registry of Australia and New Zealand (BRANZ) over this period: The Alfred, Fiona Stanley Hospital, Royal North Shore Hospital, Concord Repatriation General Hospital, Royal Darwin Hospital, Royal Adelaide Hospital, Royal Brisbane and Women’s Hospital, and Royal Hobart Hospital. Each contributing burn service was randomly assigned a letter value (i.e., A – H) to prevent individual services being identified.

Age at the time of injury was presented as a continuous variable after being calculated from date of birth and injury data. Gender was recoded to a binary variable for male gender. The primary cause of the burn injury was simplified to flame, scald, contact and other cause (e.g., friction, scald, contact, electricity, etc.). The time from injury to admission to a specialist burn service was calculated using date and time of injury and admission data. The referral source (i.e., how patient arrived at BRANZ hospital) was simplified to: direct from scene of injury via an ambulance, referred via another hospital, referred via outpatients department, or via another source (i.e., self-presentations without a referral where the patients transported themselves to hospital, referred via general practitioner, primary care physician, or other unclassified referral sources). The time from injury to first skin grafting episode was calculated using date and time of injury and surgical procedure data. Disposition status was recoded to identify patients who survived to discharge, and those who died during their admission. For patients surviving to discharge, disposition was categorised as either home or usual residence (where patient normally lived prior to their burn), another hospital or healthcare facility (including other specialist burn services, hospitals without a specialist burn services, psychiatric hospitals or services, and other rehabilitation hospitals) or other disposition (e.g., left against medical advice, etc.). Hospital length of stay (LOS) was calculated using date and time of admission and discharge data. The percentage of total body surface area (%TBSA) burned served as the primary measure of burn severity. Burns affecting ≥ 20% TBSA were deemed major burns. The LOS/%TBSA (in days) was calculated using LOS and %TBSA data.

Figures were produced in the R statistical environment version 4.0.3^1^ using the tidyverse,^2^ cowplot,^3^ ggsci,^4^ and RColorBrewer^5^ packages.

1. R Development Core Team. R: A language and environment for statistical computing. *R Foundation for Statistical Computing*. Vienna, Austria, 2014. <http://www.R-project.org/>.

2. Wickham H. tidyverse: Easily Install and Load the 'Tidyverse'. 2017; R package version 1.2.1. <https://CRAN.R-project.org/package=tidyverse>.

3. Wilke CO. cowplot: Streamlined Plot Theme and Plot Annotations for 'ggplot2'. 2019; R package version 0.9.4. <https://CRAN.R-project.org/package=cowplot>.

4. Xiao N. ggsci: Scientific Journal and Sci-Fi Themed Color Palettes for 'ggplot2'. 2018; R package version 2.9. <https://CRAN.R-project.org/package=ggsci>.

5. Neuwirth E. RColorBrewer: ColorBrewer Palettes. 2014; R package version 1.1-2. <https://CRAN.R-project.org/package=RColorBrewer>.
