# Supplementary material for: Examining the patient profile and variance of management and in‐hospital outcomes for Australian adult burns patients
Source: ANZ J Surg. 2022 Aug 22;92(10):2641–7. doi: 10.1111/ans.17985 (PMC9804322; doi:10.1111/ans.17985)
Supplement: Supplementary file 2 — Document S2: Description of patient population and demographic, injury, and clinical measure variation between services. [file ANS-92-2641-s006.docx]

**Document S2 – Description of patient population and demographic, injury, and clinical measure variation between services**

The number of admissions at each service over the four-year study period ranged from 242 to 1599 (Table 1). Patients at services C and H were older than patients at service F (Table S7). Services D, G, and H admitted a greater proportion of male patients than services B and C (Table S8). Service H had the greatest proportion of patients sustaining a flame burn; service C had the greatest proportion of scalds; service F had the greatest proportion of burns from other causes (e.g., friction, electrical, etc.; Table S9). Service A had a smaller median TBSA burned than all other services besides service B, while services E and H had larger median TBSA burned compared to most services (Table S10). Services A and C had a smaller proportion of patients with major burns compared to services E, G, and H (Table S11). Services A and C had a smaller proportion of patients with an inhalation injury (Table S12). Service A had the smallest proportion of patients with only superficial burns; service G had the greatest proportion of full thickness burns (Table S13). Service H had the shortest median time to admission apart from service F (Table S14). Service E had a smaller proportion of patients arriving direct to hospital from the scene and a greater proportion of patients referred via another hospital (Table S15). Services A and B had a greater proportion of patients admitted via outpatients; service H had a greater proportion of patients arriving direct from the scene. Service F had a smaller proportion of patients mechanically ventilated in ICU compared to services E, G, and H (Table S16). Service E had a shorter median ventilation time compared to service G (Table S17). Services A, C, and D had a greater proportion of patients discharged to home (Table S18). Service H had a greater proportion of patients discharged to another hospital or healthcare.
