# Supplementary material for: Examining the patient profile and variance of management and in‐hospital outcomes for Australian adult burns patients
Source: ANZ J Surg. 2022 Aug 22;92(10):2641–7. doi: 10.1111/ans.17985 (PMC9804322; doi:10.1111/ans.17985)
Supplement: Supplementary file 3 — Figure S1: Patient flow chart and characteristics. [file ANS-92-2641-s001.docx]

*
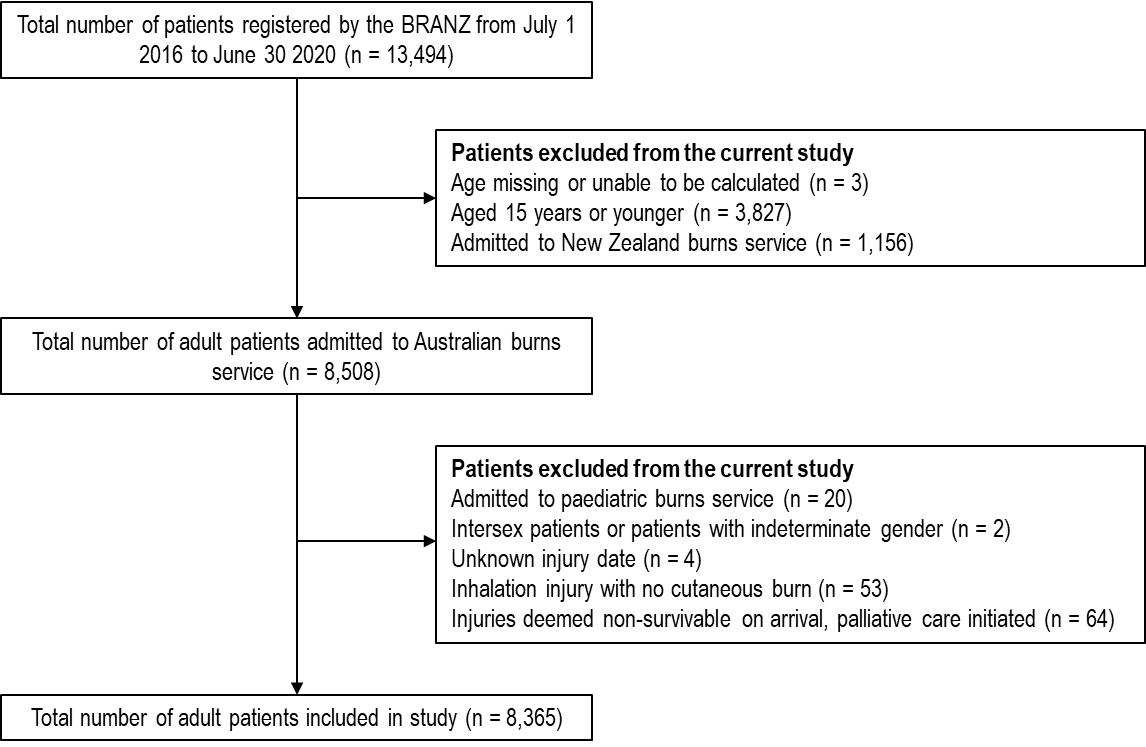
*

**Figure S1.** Patient flow chart and characteristics. BRANZ = Burns Registry of Australia and New Zealand; ICU = intensive care unit; LOS = length of stay; TBSA = total body surface area.
