# Supplementary material for: Examining the patient profile and variance of management and in‐hospital outcomes for Australian adult burns patients
Source: ANZ J Surg. 2022 Aug 22;92(10):2641–7. doi: 10.1111/ans.17985 (PMC9804322; doi:10.1111/ans.17985)
Supplement: Supplementary file 4 — Figure S2: Year‐by‐year changes for (a) ICU admissions, (b) skin grafts, (c) median LOS, (d) median LOS/TBSA burned, (e) in‐hospital mortality, and (f) unplanned readmissions at individual service level. [file ANS-92-2641-s002.docx]

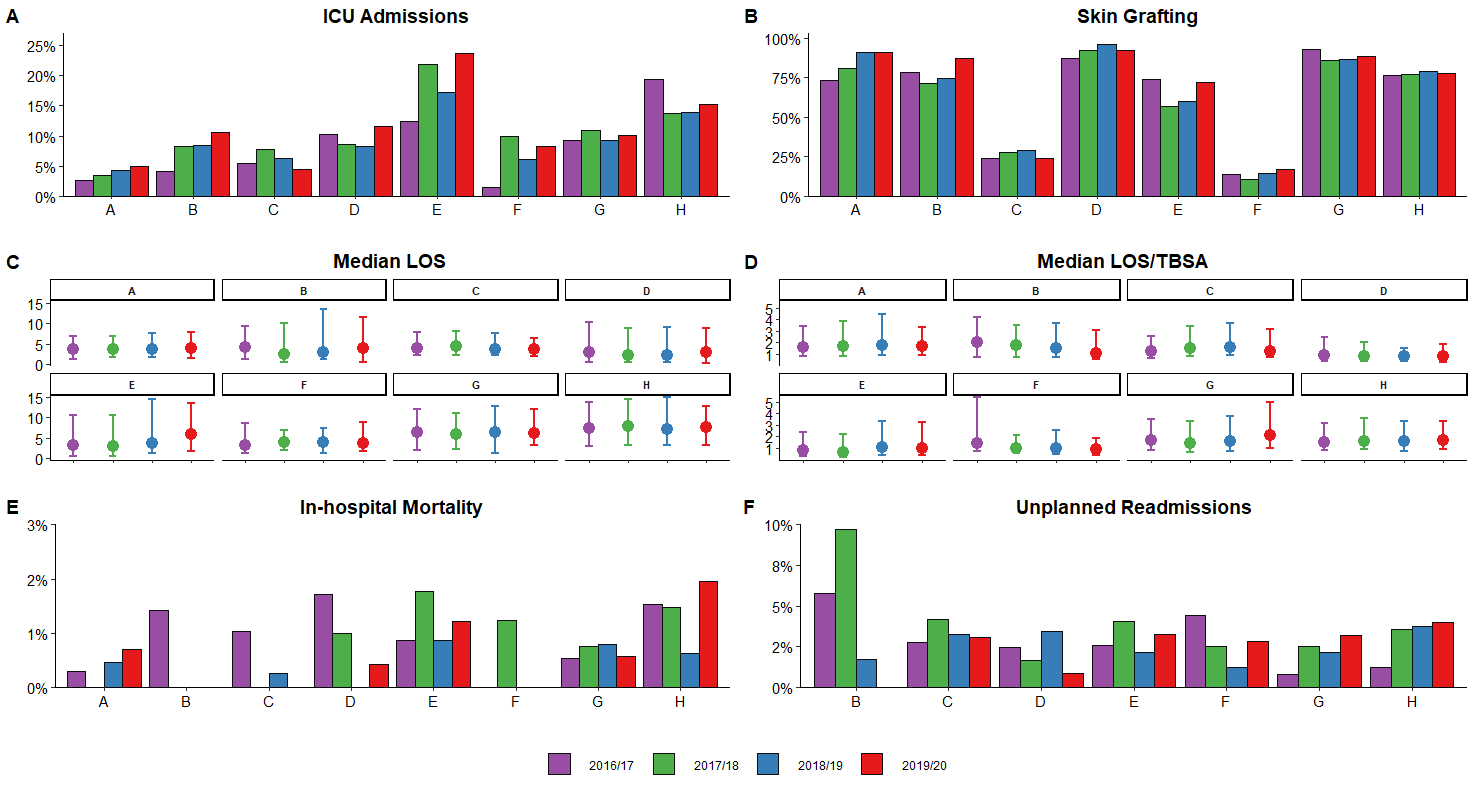


**Figure S2.** Year-by-year changes for (A) ICU admissions, (B) skin grafts, (C) median LOS, (D) median LOS/TBSA burned, (E) in-hospital mortality, and (F) unplanned readmissions at individual service level**.** ICU = intensive care unit, LOS = length of stay, TBSA = total body surface area. Percentage of patients receiving skin graft is relative to number of patients undergoing a burn wound management in theatre. LOS and LOS/TBSA data presented as median and interquartile range.
