# Supplementary material for: Examining the patient profile and variance of management and in‐hospital outcomes for Australian adult burns patients
Source: ANZ J Surg. 2022 Aug 22;92(10):2641–7. doi: 10.1111/ans.17985 (PMC9804322; doi:10.1111/ans.17985)
Supplement: Supplementary file 5 — Figure S3: Regression modelling output for selected clinical measures of interest. (a) Adjusted mean length of stay, (b) adjusted proportion of in‐hospital mortality, and (c) adjusted proportion of patients experiencing unplanned readmission. [file ANS-92-2641-s033.docx]

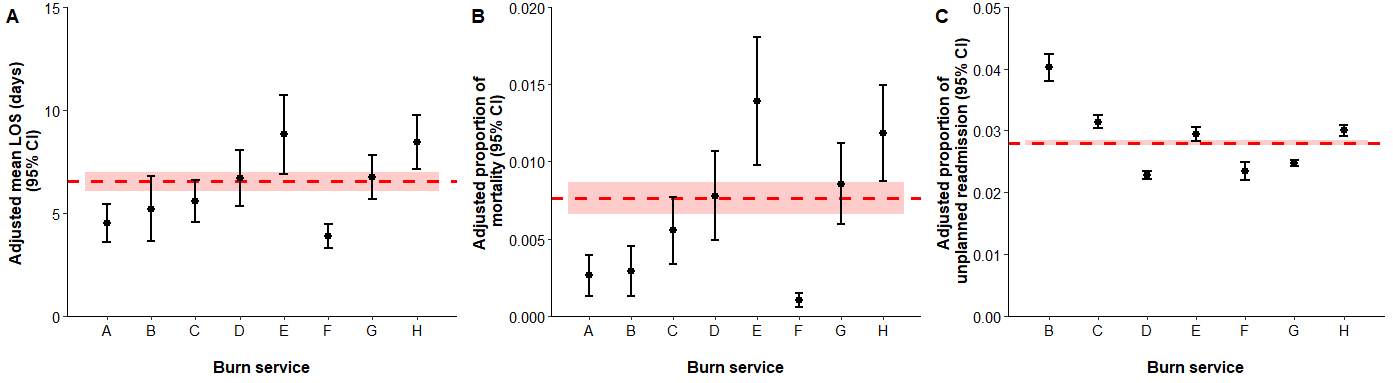


**Figure S3.** Regression modelling output for selected clinical measures of interest. (A) Adjusted mean length of stay (LOS), (B) adjusted proportion of in-hospital mortality, and (C) adjusted proportion of patients experiencing unplanned readmission. Error bars represent 95% confidence intervals (CI). The red dotted line represents the overall proportion/mean for the whole sample; the red shading represents the overall 95% CI. The adjusted proportions and means account for the random effect of contributing burn service and the fixed effects of the following covariates: percentage total body surface area, maximum recorded burn depth, age, gender, the presence of an inhalation injury, the primary cause of the injury, whether a special body area (i.e., face, hands, feet, or genitals/perineum) was affected, and whether the patient was admitted to intensive care. Whether the patient received a skin graft was not included as a covariate; this variable was omitted from the regression models due to collinearity.
