# Supplementary material for: Examining the patient profile and variance of management and in‐hospital outcomes for Australian adult burns patients
Source: ANZ J Surg. 2022 Aug 22;92(10):2641–7. doi: 10.1111/ans.17985 (PMC9804322; doi:10.1111/ans.17985)
Supplement: Supplementary file 6 — Table S1: Modelling output for adjusted proportion of admission to ICU. [file ANS-92-2641-s022.docx]

| **Table S1:** Modelling output for adjusted proportion of admission to ICU | | |
| --- | --- | --- |
|  | **Coefficient (95% CI)** | ***p*** |
| Age | 0.01 (0, 0.01) | 0.02 |
| Gender |  |  |
| Male (reference) | 1 |  |
| Female | 0.2 (-0.1, 0.4) | 0.16 |
| TBSA | 0.1 (0.1, 0.1) | <0.001 |
| Inhalation injury | 4.2 (3.8, 4.7) | <0.001 |
| Burn cause |  |  |
| Flame (reference) | 1 |  |
| Scald | -1.3 (-1.7, -1.0) | <0.001 |
| Contact | -0.9 (-1.4, -0.4) | <0.001 |
| Other cause | -0.3 (-0.6, 0.1) | 0.13 |
| Special body area burned | 0.7 (0.5, 1.0) | <0.001 |
| Deepest skin layer affected |  |  |
| Superficial dermal (reference) | 1 |  |
| Mid dermal | -0.4 (-0.7, 0) | 0.04 |
| Deep dermal | -0.3 (-0.7, 0.1) | 0.09 |
| Full thickness | 0.3 (0, 0.6) | 0.07 |
| CI = confidence interval; ICU = intensive care unit; TBSA = total body surface area. | | |
