# Supplementary material for: Examining the patient profile and variance of management and in‐hospital outcomes for Australian adult burns patients
Source: ANZ J Surg. 2022 Aug 22;92(10):2641–7. doi: 10.1111/ans.17985 (PMC9804322; doi:10.1111/ans.17985)
Supplement: Supplementary file 7 — Table S2: Modelling output for adjusted proportion of receiving skin graft. [file ANS-92-2641-s005.docx]

| **Table S2:** Modelling output for adjusted proportion of receiving skin graft | | |
| --- | --- | --- |
|  | **Coefficient (95% CI)** | ***p*** |
| Age | 0.01 (0.01, 0.01) | <0.001 |
| Gender |  | 0.51 |
| Male (reference) | 1 |  |
| Female | -0.06 (-0.22, 0.11) |  |
| TBSA | -0.02 (-0.03, -0.02) | <0.001 |
| Inhalation injury | -0.40 (-0.79, -0.01) | 0.04 |
| Burn cause |  |  |
| Flame (reference) | 1 |  |
| Scald | 0.09 (-0.10, 0.28) | 0.36 |
| Contact | -0.12 (-0.36, 0.11) | 0.31 |
| Other cause | -0.29 (-0.53, -0.05) | 0.02 |
| Special body area burned | -0.03 (-0.19, 0.12) | 0.73 |
| Deepest skin layer affected |  |  |
| Superficial dermal (reference) | 1 |  |
| Mid dermal | 0.44 (0.15, 0.72) | 0.003 |
| Deep dermal | 2.43 (2.15, 2.70) | <0.001 |
| Full thickness | 3.07 (2.79, 3.34) | <0.001 |
| CI = confidence interval; TBSA = total body surface area. | | |
