# Supplementary material for: Examining the patient profile and variance of management and in‐hospital outcomes for Australian adult burns patients
Source: ANZ J Surg. 2022 Aug 22;92(10):2641–7. doi: 10.1111/ans.17985 (PMC9804322; doi:10.1111/ans.17985)
Supplement: Supplementary file 8 — Table S3: Modelling output for adjusted mean LOS. [file ANS-92-2641-s018.docx]

| **Table S3:** Modelling output for adjusted mean LOS | | |
| --- | --- | --- |
|  | **Coefficient (95% CI)** | ***p*** |
| Age | 0.02 (0.02, 0.02) | <0.001 |
| Gender |  | <0.001 |
| Male (reference) | 1 |  |
| Female | -0.13 (-0.19, -0.07) |  |
| TBSA | 0.06 (0.05, 0.06) | <0.001 |
| Inhalation injury | 0.02 (-0.13, 0.16) | 0.83 |
| Burn cause |  |  |
| Flame (reference) | 1 |  |
| Scald | -0.22 (-0.29, -0.15) | <0.001 |
| Contact | -0.38 (-0.46, -0.29) | <0.001 |
| Other cause | -0.23 (-0.32, -0.15) | <0.001 |
| Special body area burned | 0.18 (0.13, 0.24) | <0.001 |
| Deepest skin layer affected |  |  |
| Superficial dermal (reference) | 1 |  |
| Mid dermal | 0.14 (0.05, 0.24) | 0.002 |
| Deep dermal | 0.27 (0.18, 0.36) | <0.001 |
| Full thickness | 0.41 (0.33, 0.50) | <0.001 |
| CI = confidence interval; LOS = length of stay; TBSA = total body surface area. | | |
