# Supplementary material for: Examining the patient profile and variance of management and in‐hospital outcomes for Australian adult burns patients
Source: ANZ J Surg. 2022 Aug 22;92(10):2641–7. doi: 10.1111/ans.17985 (PMC9804322; doi:10.1111/ans.17985)
Supplement: Supplementary file 9 — Table S4: Modelling output for adjusted mean LOS/TBSA. [file ANS-92-2641-s023.docx]

| **Table S4:** Modelling output for adjusted mean LOS/TBSA | | |
| --- | --- | --- |
|  | **Coefficient (95% CI)** | ***p*** |
| Age | 0.02 (0.01, 0.02) | <0.001 |
| Gender |  | 0.95 |
| Male (reference) | 1 |  |
| Female | 0 (-0.01, 0.01) |  |
| Inhalation injury | -0.24 (-0.38, -0.11) | <0.001 |
| Burn cause |  |  |
| Flame (reference) | 1 |  |
| Scald | 0.28 (0.22, 0.35) | <0.001 |
| Contact | 0.67 (0.59, 0.75) | <0.001 |
| Other cause | 0.62 (0.53, 0.70) | <0.001 |
| Special body area burned | 0.31 (0.26, 0.37) | <0.009 |
| Deepest skin layer affected |  |  |
| Superficial dermal (reference) | 1 |  |
| Mid dermal | 0.08 (-0.01, 0.17) | 0.09 |
| Deep dermal | 0.35 (0.26, 0.45) | <0.001 |
| Full thickness | 0.68 (0.60, 0.77) | <0.001 |
| CI = confidence interval; LOS = length of stay; TBSA = total body surface area. | | |
