# Supplementary material for: Examining the patient profile and variance of management and in‐hospital outcomes for Australian adult burns patients
Source: ANZ J Surg. 2022 Aug 22;92(10):2641–7. doi: 10.1111/ans.17985 (PMC9804322; doi:10.1111/ans.17985)
Supplement: Supplementary file 10 — Table S5: Modelling output for adjusted proportion of in‐hospital mortality. [file ANS-92-2641-s012.docx]

| **Table S5:** Modelling output for adjusted proportion of in-hospital mortality | | |
| --- | --- | --- |
|  | **Coefficient (95% CI)** | ***p*** |
| Age | 0.08 (0.06, 0.10) | <0.001 |
| Gender |  | 0.39 |
| Male (reference) | 1 |  |
| Female | 0.33 (-0.38, 1.03) |  |
| TBSA | 0.07 (0.06, 0.09) | <0.001 |
| Inhalation injury | 1.36 (0.59, 2.13) | 0.001 |
| Burn cause |  |  |
| Flame (reference) | 1 |  |
| Scald | -0.12 (-1.15, 0.90) | 0.82 |
| Contact | 0.35 (-0.79, 1.49) | 0.55 |
| Other cause | -1.12 (-3.22, 0.97) | 0.29 |
| Special body area burned | 0.52 (-0.39, 1.42) | 0.26 |
| Deepest skin layer affected |  |  |
| Superficial dermal (reference) | 1 |  |
| Mid dermal | 1.00 (-1.35, 3.34) | 0.41 |
| Deep dermal | 1.39 (-0.87, 3.64) | 0.23 |
| Full thickness | 2.10 (-0.02, 4.22) | 0.05 |
| CI = confidence interval; TBSA = total body surface area. | | |
