# Supplementary material for: Examining the patient profile and variance of management and in‐hospital outcomes for Australian adult burns patients
Source: ANZ J Surg. 2022 Aug 22;92(10):2641–7. doi: 10.1111/ans.17985 (PMC9804322; doi:10.1111/ans.17985)
Supplement: Supplementary file 11 — Table S6: Modelling output for adjusted proportion of unplanned readmissions. [file ANS-92-2641-s008.docx]

| **Table S6:** Modelling output for adjusted proportion of unplanned readmissions | | |
| --- | --- | --- |
|  | **Coefficient (95% CI)** | ***p*** |
| Age | 0 (-0.01, 0.01) | 0.96 |
| Gender |  | 0.20 |
| Male (reference) | 1 |  |
| Female | 0.21 (-0.11, 0.53) |  |
| TBSA | 0.02 (0.01, .03) | 0.003 |
| Inhalation injury | -1.19 (-2.18, -0.19) | 0.02 |
| Burn cause |  |  |
| Flame (reference) | 1 |  |
| Scald | 0.05 (-0.35, 0.46) | 0.80 |
| Contact | 0.37 (-0.07, 0.82) | 0.10 |
| Other cause | 0.29 (-0.19, 0.76) | 0.24 |
| Special body area burned | 0.24 (-0.08, 0.57) | 0.14 |
| Deepest skin layer affected |  |  |
| Superficial dermal (reference) | 1 |  |
| Mid dermal | -0.68 (-1.31, -0.04) | 0.04 |
| Deep dermal | 0.24 (-0.29, 0.76) | 0.38 |
| Full thickness | 0.60 (0.12, 1.08) | 0.01 |
| CI = confidence interval; TBSA = total body surface area. | | |
