# Supplementary material for: Examining the patient profile and variance of management and in‐hospital outcomes for Australian adult burns patients
Source: ANZ J Surg. 2022 Aug 22;92(10):2641–7. doi: 10.1111/ans.17985 (PMC9804322; doi:10.1111/ans.17985)
Supplement: Supplementary file 12 — Table S7: Pairwise comparisons for age by service. [file ANS-92-2641-s030.docx]

| **Table S7:** Pairwise comparisons for age by service | | | | | | | |
| --- | --- | --- | --- | --- | --- | --- | --- |
|  | A | B | C | D | E | F | G |
| B | 0.99 |  |  |  |  |  |  |
| C | 0.004 | 0.99 |  |  |  |  |  |
| D | 0.99 | 0.99 | 0.42 |  |  |  |  |
| E | 0.38 | 0.99 | 0.99 | 0.99 |  |  |  |
| F | 0.69 | 0.99 | **<0.001** | 0.14 | 0.02 |  |  |
| G | 0.99 | 0.99 | 0.003 | 0.99 | 0.30 | 0.85 |  |
| H | 0.006 | 0.99 | 0.99 | 0.53 | 0.99 | **<0.001** | 0.004 |
| Data presented as *p*-values. **Bold** text represents significant pairwise comparisons after Bonferroni correction for multiple comparisons. | | | | | | | |
