# Supplementary material for: Examining the patient profile and variance of management and in‐hospital outcomes for Australian adult burns patients
Source: ANZ J Surg. 2022 Aug 22;92(10):2641–7. doi: 10.1111/ans.17985 (PMC9804322; doi:10.1111/ans.17985)
Supplement: Supplementary file 13 — Table S8: Pairwise comparisons for male gender by service. [file ANS-92-2641-s004.docx]

| **Table S8:** Pairwise comparisons for male gender by service | | | | | | | |
| --- | --- | --- | --- | --- | --- | --- | --- |
|  | A | B | C | D | E | F | G |
| B | 0.006 |  |  |  |  |  |  |
| C | 0.07 | 0.08 |  |  |  |  |  |
| D | 0.04 | **<0.001** | **<0.001** |  |  |  |  |
| E | 0.76 | 0.005 | 0.07 | 0.12 |  |  |  |
| F | 0.19 | 0.002 | 0.02 | 0.97 | 0.28 |  |  |
| G | 0.02 | **<0.001** | **<0.001** | 0.94 | 0.08 | 0.99 |  |
| H | 0.04 | **<0.001** | **<0.001** | 0.93 | 0.12 | 0.93 | 0.86 |
| Data presented as *p*-values. **Bold** text represents significant pairwise comparisons after Bonferroni correction for multiple comparisons. | | | | | | | |
