# Supplementary material for: Examining the patient profile and variance of management and in‐hospital outcomes for Australian adult burns patients
Source: ANZ J Surg. 2022 Aug 22;92(10):2641–7. doi: 10.1111/ans.17985 (PMC9804322; doi:10.1111/ans.17985)
Supplement: Supplementary file 16 — Table S11: Pairwise comparisons for major burns by service. [file ANS-92-2641-s028.docx]

| **Table S11:** Pairwise comparisons for major burns by service | | | | | | | |
| --- | --- | --- | --- | --- | --- | --- | --- |
|  | A | B | C | D | E | F | G |
| B | 0.26 |  |  |  |  |  |  |
| C | 0.40 | 0.52 |  |  |  |  |  |
| D | 0.009 | 0.70 | 0.08 |  |  |  |  |
| E | **<0.001** | 0.05 | **<0.001** | 0.006 |  |  |  |
| F | 0.66 | 0.60 | 0.97 | 0.30 | 0.01 |  |  |
| G | **<0.001** | 0.14 | **<0.001** | 0.05 | 0.31 | 0.03 |  |
| H | **<0.001** | 0.06 | **<0.001** | 0.006 | 0.83 | 0.01 | 0.38 |
| Data presented as *p*-values. **Bold** text represents significant pairwise comparisons after Bonferroni correction for multiple comparisons. | | | | | | | |
