# Supplementary material for: Examining the patient profile and variance of management and in‐hospital outcomes for Australian adult burns patients
Source: ANZ J Surg. 2022 Aug 22;92(10):2641–7. doi: 10.1111/ans.17985 (PMC9804322; doi:10.1111/ans.17985)
Supplement: Supplementary file 21 — Table S16: Pairwise comparisons for mechanical ventilation in ICU by service. [file ANS-92-2641-s011.docx]

| **Table S16:** Pairwise comparisons for mechanical ventilation in ICU by service | | | | | | | |
| --- | --- | --- | --- | --- | --- | --- | --- |
|  | A | B | C | D | E | F | G |
| B | 0.14 |  |  |  |  |  |  |
| C | 0.90 | 0.11 |  |  |  |  |  |
| D | 0.91 | 0.14 | 0.78 |  |  |  |  |
| E | 0.07 | 0.53 | 0.02 | 0.03 |  |  |  |
| F | 0.04 | 0.004 | 0.03 | 0.02 | **<0.001** |  |  |
| G | 0.007 | 0.90 | **0.001** | 0.002 | 0.23 | **<0.001** |  |
| H | 0.04 | 0.60 | 0.008 | 0.01 | 0.78 | **<0.001** | 0.34 |
| Data presented as *p*-values. **Bold** text represents significant pairwise comparisons after Bonferroni correction for multiple comparisons. ICU = intensive care unit. | | | | | | | |
