# Supplementary material for: Examining the patient profile and variance of management and in‐hospital outcomes for Australian adult burns patients
Source: ANZ J Surg. 2022 Aug 22;92(10):2641–7. doi: 10.1111/ans.17985 (PMC9804322; doi:10.1111/ans.17985)
Supplement: Supplementary file 28 — Table S23: Pairwise comparisons for adjusted proportion of patients receiving a skin graft. [file ANS-92-2641-s009.docx]

| **Table S23:** Pairwise comparisons for adjusted proportion of patients receiving a skin graft | | | | | | | |
| --- | --- | --- | --- | --- | --- | --- | --- |
|  | A | B | C | D | E | F | G |
| B | **<0.001** |  |  |  |  |  |  |
| C | **<0.001** | **<0.001** |  |  |  |  |  |
| D | **<0.001** | **<0.001** | **<0.001** |  |  |  |  |
| E | **<0.001** | **<0.001** | **<0.001** | **<0.001** |  |  |  |
| F | **<0.001** | **<0.001** | **<0.001** | **<0.001** | **<0.001** |  |  |
| G | **<0.001** | **<0.001** | **<0.001** | **<0.001** | **<0.001** | **<0.001** |  |
| H | **<0.001** | 0.66 | **<0.001** | **<0.001** | **<0.001** | **<0.001** | **<0.001** |
| Data presented as *p*-values. **Bold** text represents significant pairwise comparisons after Bonferroni correction for multiple comparisons. | | | | | | | |
