# Supplementary material for: Examining the patient profile and variance of management and in‐hospital outcomes for Australian adult burns patients
Source: ANZ J Surg. 2022 Aug 22;92(10):2641–7. doi: 10.1111/ans.17985 (PMC9804322; doi:10.1111/ans.17985)
Supplement: Supplementary file 29 — Table S24: Pairwise comparisons for hospital LOS by service. [file ANS-92-2641-s034.docx]

| **Table S24:** Pairwise comparisons for hospital LOS by service | | | | | | | |
| --- | --- | --- | --- | --- | --- | --- | --- |
|  | A | B | C | D | E | F | G |
| B | 0.99 |  |  |  |  |  |  |
| C | 0.002 | 0.99 |  |  |  |  |  |
| D | 0.08 | 0.76 | **<0.001** |  |  |  |  |
| E | 0.015 | 0.99 | 0.99 | **<0.001** |  |  |  |
| F | 0.99 | 0.99 | 0.99 | 0.06 | 0.99 |  |  |
| G | **<0.001** | **<0.001** | **<0.001** | **<0.001** | **<0.001** | **<0.001** |  |
| H | **<0.001** | **<0.001** | **<0.001** | **<0.001** | **<0.001** | **<0.001** | **<0.001** |
| Data presented as *p*-values. **Bold** text represents significant pairwise comparisons after Bonferroni correction for multiple comparisons. LOS = length of stay. | | | | | | | |
