# Supplementary material for: Examining the patient profile and variance of management and in‐hospital outcomes for Australian adult burns patients
Source: ANZ J Surg. 2022 Aug 22;92(10):2641–7. doi: 10.1111/ans.17985 (PMC9804322; doi:10.1111/ans.17985)
Supplement: Supplementary file 30 — Table S25: Pairwise comparisons for adjusted mean LOS. [file ANS-92-2641-s031.docx]

| **Table S25:** Pairwise comparisons for adjusted mean LOS | | | | | | | |
| --- | --- | --- | --- | --- | --- | --- | --- |
|  | A | B | C | D | E | F | G |
| B | 0.74 |  |  |  |  |  |  |
| C | 0.03 | 0.45 |  |  |  |  |  |
| D | 0.04 | 0.42 | 0.91 |  |  |  |  |
| E | **<0.001** | 0.10 | 0.11 | 0.17 |  |  |  |
| F | 0.80 | 0.65 | 0.16 | 0.16 | 0.02 |  |  |
| G | 0.005 | 0.28 | 0.54 | 0.64 | 0.30 | 0.08 |  |
| H | **0.001** | 0.15 | 0.19 | 0.26 | 0.70 | 0.03 | 0.47 |
| Data presented as *p*-values. **Bold** text represents significant pairwise comparisons after Bonferroni correction for multiple comparisons. LOS = length of stay. | | | | | | | |
