# Supplementary material for: Examining the patient profile and variance of management and in‐hospital outcomes for Australian adult burns patients
Source: ANZ J Surg. 2022 Aug 22;92(10):2641–7. doi: 10.1111/ans.17985 (PMC9804322; doi:10.1111/ans.17985)
Supplement: Supplementary file 31 — Table S26: Pairwise comparisons for adjusted mean LOS/TBSA burned. [file ANS-92-2641-s025.docx]

| **Table S26:** Pairwise comparisons for adjusted mean LOS/TBSA burned | | | | | | | |
| --- | --- | --- | --- | --- | --- | --- | --- |
|  | A | B | C | D | E | F | G |
| B | 0.004 |  |  |  |  |  |  |
| C | 0.51 | **0.001** |  |  |  |  |  |
| D | 0.05 | 0.08 | 0.01 |  |  |  |  |
| E | **0.001** | 0.32 | **<0.001** | 0.23 |  |  |  |
| F | 0.28 | 0.002 | 0.47 | 0.03 | 0.003 |  |  |
| G | **<0.001** | 0.12 | **<0.001** | **<0.001** | **<0.001** | **<0.001** |  |
| H | 0.004 | **<0.001** | 0.03 | **<0.001** | **<0.001** | 0.63 | **<0.001** |
| Data presented as *p*-values. **Bold** text represents significant pairwise comparisons after Bonferroni correction for multiple comparisons. LOS = length of stay, TBSA = total body surface area. | | | | | | | |
