# Supplementary material for: Examining the patient profile and variance of management and in‐hospital outcomes for Australian adult burns patients
Source: ANZ J Surg. 2022 Aug 22;92(10):2641–7. doi: 10.1111/ans.17985 (PMC9804322; doi:10.1111/ans.17985)
Supplement: Supplementary file 33 — Table S28: Pairwise comparisons for adjusted proportion of in‐hospital mortality. [file ANS-92-2641-s013.docx]

| **Table S28:** Pairwise comparisons for adjusted proportion of in-hospital mortality | | | | | | | |
| --- | --- | --- | --- | --- | --- | --- | --- |
|  | A | B | C | D | E | F | G |
| B | 0.88 |  |  |  |  |  |  |
| C | 0.18 | 0.60 |  |  |  |  |  |
| D | 0.02 | 0.24 | 0.24 |  |  |  |  |
| E | **<0.001** | 0.20 | **0.001** | 0.05 |  |  |  |
| F | 0.61 | 0.62 | 0.22 | 0.06 | 0.002 |  |  |
| G | 0.002 | 0.16 | 0.09 | 0.70 | 0.09 | 0.03 |  |
| H | **<0.001** | 0.01 | **<0.001** | 0.02 | 0.76 | **0.001** | 0.03 |
| Data presented as *p*-values. **Bold** text represents significant pairwise comparisons after Bonferroni correction for multiple comparisons. | | | | | | | |
