# Supplementary material for: Examining the patient profile and variance of management and in‐hospital outcomes for Australian adult burns patients
Source: ANZ J Surg. 2022 Aug 22;92(10):2641–7. doi: 10.1111/ans.17985 (PMC9804322; doi:10.1111/ans.17985)
Supplement: Supplementary file 34 — Table S29: Pairwise comparisons for predicted probability of unplanned readmission. [file ANS-92-2641-s019.docx]

| **Table S29:** Pairwise comparisons for predicted probability of unplanned readmission | | | | | | | |
| --- | --- | --- | --- | --- | --- | --- | --- |
|  | A | B | C | D | E | F | G |
| B | --- |  |  |  |  |  |  |
| C | --- | **<0.001** |  |  |  |  |  |
| D | --- | **<0.001** | **<0.001** |  |  |  |  |
| E | --- | **<0.001** | 0.01 | **<0.001** |  |  |  |
| F | --- | **<0.001** | **<0.001** | 0.72 | **<0.001** |  |  |
| G | --- | **<0.001** | **<0.001** | 0.003 | **<0.001** | 0.17 |  |
| H | --- | **<0.001** | 0.06 | **<0.001** | 0.45 | **<0.001** | **<0.001** |
| Data presented as *p*-values. **Bold** text represents significant pairwise comparisons after Bonferroni correction for multiple comparisons. Readmissions data not available for service A. | | | | | | | |
